# A Case of Polydactyly in a Roe Deer ( *Capreolus capreolus* ) From Germany

**DOI:** 10.1111/ahe.70164

**Published:** 2026-08-07

**Authors:** Johannes Lang, Sarah P. Stubbe, Carsten Staszyk, Michael Lierz

**Affiliations:** ^1^ Clinic for Birds, Reptiles, Amphibians and Fish, Working Group for Wildlife Research Justus‐Liebig‐University Giessen Giessen Germany; ^2^ Institute for Veterinary Anatomy, Histology, and Embryology Justus‐Liebig‐University Giessen Giessen Germany

**Keywords:** Cervidae, congenital anomaly, metatarsus, supernumerary digits

## Abstract

Polydactyly is a rare anomaly that has been described in many vertebrate species including several deer species. The case described in this communication is believed to be only the second documented example of additional metatarsae in roe deer ( *Capreolus capreolus* ). In addition to normally developed digits III and IV and the dewclaws, a right hind foot of an adult female from Germany had two additional digits lateral to digit V. Both originated from additional metatarsal elements. This case offers significant insights into the developmental plasticity of the cervid limb and extends the morphological spectrum of cervid polydactyly.

## 1 Introduction

Polydactyly, the presence of one or more supernumerary digits, is a rare congenital anomaly that has been described in a wide range of vertebrates, including domestic mammals and birds, laboratory animals and humans (Al‐Ani et al. 1998; Clark et al. 2000; Frey et al. 2001; Bahr et al. 2003; Fayeye et al. 2006; Sakai 2006). Supernumerary digits in animals may be of teratologic or developmental (atavistic) origin; inherited cases are commonly associated with bilateral symmetry (Stanek and Hantak 1986). In wild ungulates, polydactyly is less common than in domestic ungulates (Chapman 2006). Among ungulates, polydactyly has been reported in several deer species worldwide.

Most documented cases of polydactyly in deer originate from New Zealand and Australia. Daniel (1967) described 90 red deer ( *Cervus elaphus* ) with supernumerary digits on the forefeet, while Davidson (1971) reported a single case in sika deer ( *Cervus nippon* ). Banwell (1999) later summarized 64 additional examples from these regions, predominantly in red deer but also including 16 rusa ( *Cervus timorensis* ), eight sika and two sambar ( *Cervus unicolor* ). Outside Australasia, polydactyly has been observed sporadically across several species. In North America, single cases have been described in caribou ( *Rangifer tarandus groenlandicus* ) (Miller and Broughton 1971) and white‐tailed deer ( *Odocoileus virginianus* ) (Miller and Cawley 1970). In Europe, Horsefield (1993) reported two examples in fallow deer ( *Dama dama* ) from England, and several cases have been noted in roe deer ( *Capreolus capreolus* ) from Scotland (Donaldson‐Webster 1995; Chapman 2006), Croatia (Pintur et al. 2011) and Germany (Gäbler 1961; Petersilie 1964; Braunschweig 1982; Schmid et al. 1990; Stubbe 1997; Schreiner 2026) although detailed descriptions are lacking in the latter cases.

Polydactyly in deer may occur in either sex and can affect the fore or hind limbs, either unilaterally or bilaterally (Chapman 2006). The additional digits can vary in form, appearing as duplications of functional toes (digits III and IV), as duplications of the dewclaws (digits II and V), or as reduced structures occupying the ancestral position of the first digit.

Despite the relatively broad taxonomic distribution of polydactyly in Cervidae, detailed descriptions of cases in roe deer remain limited. The aim of the present study is therefore to contribute to the limited body of knowledge on this subject by reporting a well‐documented and described case of polydactyly in a roe deer from Germany.

## 2 Case Description

**FIGURE 1:**
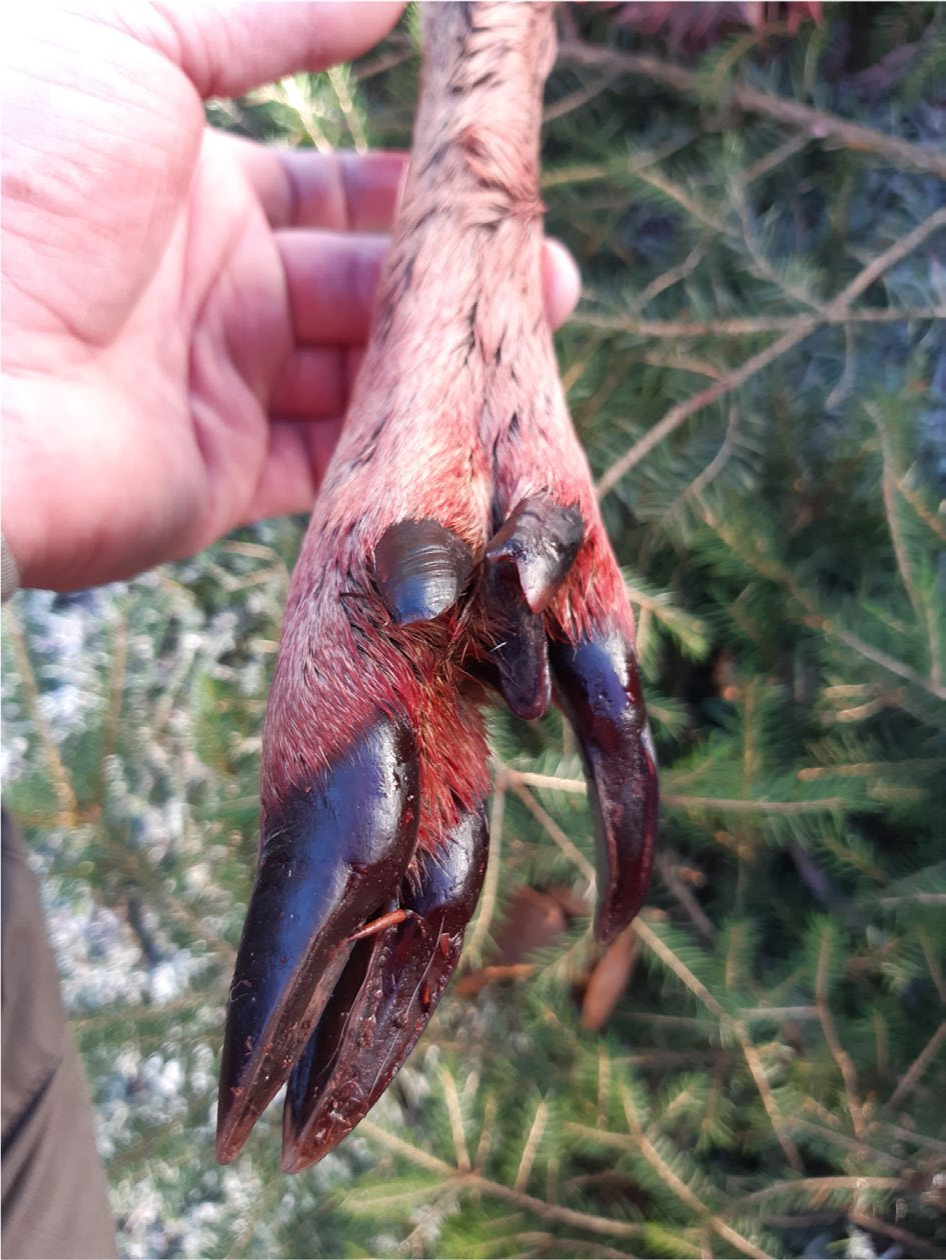
Right hind foot of an adult female roe deer with two additional digits shot in January 2020 on a drive hunt in Hesse (Germany).

The abnormal right hind foot of an adult female roe deer shot in January 2020 on a drive hunt in Hesse, Germany (50° 32′ N/8° 31′ E) was presented (Figure 1). After external examination, the foot was cleaned using dermestid beetles (Timm et al. 2020). After that, all digits were measured along the curve of the bones to the nearest mm.

**FIGURE 2:**
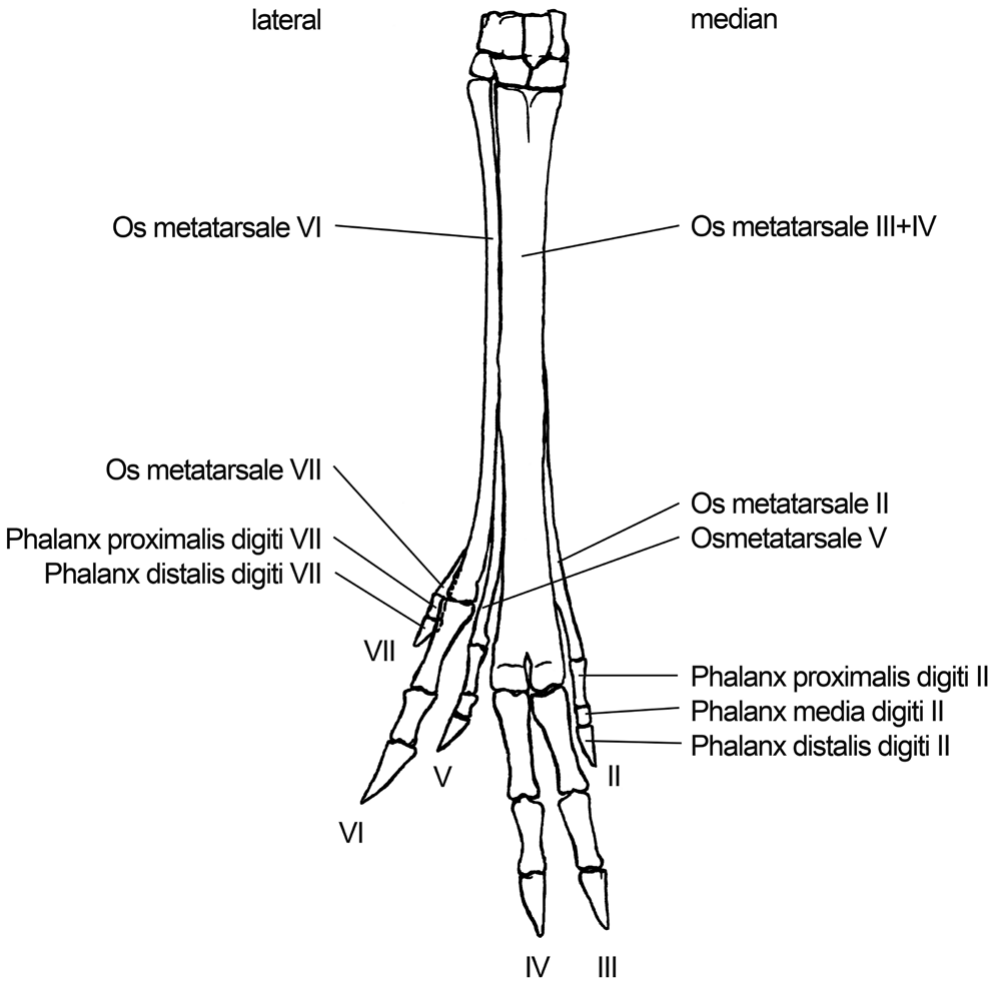
Drawing from the cleaned bones of an adult female roe deer with two additional digits shot in January 2020 on a drive hunt in Hesse (Germany), showing two additional metatarsal elements with two additional digits lateral to digit V. The anatomical nomenclature for bones on digits III to VI follows the ones mentioned at digit II, with no additional references provided for enhanced clarity.

In this specimen, the right hind foot was affected. In addition to normally developed digits III and IV and the dewclaws (digits II and V), there were two additional digits lateral to digit V: one normal digit (VI) and one dewclaw (VII). Both originated from additional metatarsal elements (Figure 2). With 139 mm, the additional metatarsus (VI) was shorter than the 154 mm long functional metatarsus (Os metatarsale III + IV). We found no bony connection of this additional metatarsus (VI) to the main metatarsus (III + IV).

The additional digit VI consisted of a complete set of three phalanges, with the proximal (24 mm), middle (16 mm) and distal (23 mm) phalanges being shorter than those of the weight‐bearing toes (30, 21 and 25 mm). The horn of the additional hoof was narrower and less worn than the weight bearing ones, while the hoof of the additional dewclaw showed excessive horn growth, with the horn growing inward. The additional dewclaw (Os metatarsale VII) had only two phalanges.

## 3 Discussion

Multiple cases of polydactyly in roe deer have been reported across Europe (Gäbler 1961; Petersilie 1964; Szabó 1966; Braunschweig 1982; Schmid et al. 1990; Oberbach 1994; Schirmer 1995; Stubbe 1997; Pintur et al. 2011; Schreiner 2026). However, many of these accounts lack comprehensive morphological descriptions or radiographic confirmation, limiting comparative interpretation.

In roe deer, the most observed form of polydactyly involves duplication of the primary functional digits (III and IV). These may appear as equally developed or reduced digits arranged either parallel to or diverging from the normal pair (Tönnies 1941; Gäbler 1961; Petersilie 1964; Schmid et al. 1990). Other variants include the duplication of dewclaws (digits II and V) (Braunschweig 1982) or the insertion of an additional digit between the normal toes (Oberbach 1994; Chapman 2006). While most cases involve a single limb, multi‐limb manifestations have also been reported occasionally (Schmid et al. 1990; Pintur et al. 2011).

An additional metacarpus has been described in one sika deer (Davidson 1971) and in red deer (Daniel 1967) from New Zealand. To the authors' knowledge, only one description of an additional metatarsus in roe deer as found in the present case can be found in the published literature to date (Pintur et al. 2011). In the case described by Davidson (1971), the additional metacarpus was significantly shorter than the functional one and had no bony connection to it. In the present case, the additional metatarsus was also shorter and was only connected to the functional metatarsus by dense connective tissue and no bony fusion was detected.

The origin of this anomaly is most likely an embryonic development disorder. While a heritable component cannot be ruled out (Daniel 1967), most evidence suggests that cervid polydactyly results from irregular duplication of digital primordia during early limb‐bud formation (Sadler 2024). Depending on the timing and location of the disturbance, this can lead to partial or complete duplication of the metacarpal or metatarsal bones and their associated phalanges. The well‐formed, structurally integrated appearance of the additional digit in this case supports a developmental origin rather than an acquired or pathological cause.

When compared to previously reported cases—such as duplication of digits III and IV in roe deer (Braunschweig 1982; Oberbach 1994) or duplication of metacarpus in roe deer and sika deer (Daniel 1967; Davidson 1971) the current example extends the morphological spectrum of cervid polydactyly.

Such cases offer significant insights into the developmental plasticity and evolutionary history of the cervid limb. Documentation and detailed morphological analysis, ideally supported by radiographic or histological data, can improve understanding of the mechanisms underlying such congenital anomalies and help identify potential genetic or environmental influences.

## Author Contributions

J.L. collected the specimen, S.P.S. performed the measurements and radiography. J.L., S.P.S., C.S. and M.L. wrote the paper together and all authors approved the final version for publication.

## Funding

The authors have nothing to report.

## Ethics Statement

The authors have nothing to report.

## Consent

The authors have nothing to report.

## Conflicts of Interest

The authors declare no conflicts of interest.

## Data Availability

The data that support the findings of this study are available from the corresponding author upon reasonable request.
